# Measurement of propagating waves from local field potentials and unit activity in the cortex of human and monkey

**DOI:** 10.1186/1471-2202-15-S1-P174

**Published:** 2014-07-21

**Authors:** Lyle E Muller, Giacomo Benvenuti, Frédéric Chavane, Alain Destexhe

**Affiliations:** 1 Unité des Neurosciences, Information et Complexité (UNIC), CNRS Gif-sur-Yvette, 91198, France; 2 Insitut de Neurosciences de la Timone (INT), Marseille, 13005, France

## 

The existence of propagating waves, either spontaneous or stimulus-evoked, in neocortex during the awake state has been a subject of recent interest [1,2]. Here, following work done previously in voltage-sensitive dye imaging of the primary visual cortex in the awake monkey [3], we apply an analysis method for non-parametric, automated detection of propagating waves in noisy multichannel data to multielectrode array (MEA) recordings taken from the neocortex of the human (middle temporal gyrus, as studied in [4]) and monkey (primary visual cortex).

In the spontaneous awake state, we detect transient, intermittent propagations occurring in the local field potential (LFP) across a wide frequency range. After selecting the propagation epochs within the highest confidence interval, we observe that the speed distribution falls naturally into the range of the horizontal fibers (0.1 - 0.5 m/s), suggesting a similar propagation substrate as that observed previously in voltage-sensitive dye imaging data [3]. With this in mind, we go on to compare the spatiotemporal dynamics on the array across various states of arousal.

One question raised by our earlier VSD analysis is the extent to which the wave evoked by a small visual stimulus is supra- or sub-threshold. Specifically, because neurons during awake, "activated" cortical states sit a few millivolts below threshold [5,6], operating in a fluctuation-driven regime [7], the transient depolarization detected in the VSD signal as the wave passes may change the background spiking probability in the local circuit. To address this, we analyzed the relationship between single- and multi-unit activity (SUA/MUA) and LFP. We study the spatiotemporal dynamics of this relationship to determine the coupling of spiking activity with transient propagating waves, and compare the results with computational models of spiking neurons.

In conclusion, we show here that a combination of multi-electrode recordings, time-series analysis, and computational modeling provides a powerful set of tools for quantifying spatiotemporal dynamics in awake states.

## References

[B1] Ray S Maunsell JH Network rhythms influence the relationship between spike-triggered local field potential and functional connectivity J Neurosci 2011 15 12674 12682 10.1523/JNEUROSCI.1856-11.2011 21880928 PMC3488382

[B2] Nauhaus I Busse L Ringrach DL Carandini M Robustness of traveling waves in ongoing activity of visual cortex J Neurosci 2012 15 3088 3094 10.1523/JNEUROSCI.5827-11.2012 22378881 PMC3436121

[B3] Muller LE Reynaud A Chavane F Destexhe A Propagating waves structure spatiotemporal activity in visual cortex of the awake monkey BMC Neurosci 2013 15 O8

[B4] Peyrache A Dehghani N Eskandar EN Madsen JR Anderson WS Donoghue JS Hochberg LR Halgren E Cash SS Destexhe A Spatiotemporal dynamics of neocortical excitation and inhibition during human sleep Proc Natl Acad Sci USA 2012 15 1731 1736 10.1073/pnas.1109895109 22307639 PMC3277175

[B5] Destexhe A Rudolph M Paré D The high-conductance state of neocortical neurons in vivo Nat Rev Neurosci 2003 15 739 751 10.1038/nrn1198 12951566

[B6] Poulet JFA Petersen CCH Internal brain state regulates membrane potential synchrony in barrel cortex of behaving mice Nature 2008 15 881 885 10.1038/nature07150 18633351

[B7] Kuhn A Aertsen A Rotter S Neuronal integration of synaptic input in the fluctuation-driven regime J Neurosci 2004 15 2345 2356 10.1523/JNEUROSCI.3349-03.2004 15014109 PMC6729484

